# Methionine-Mediated Regulation of Intestinal Lipid Transportation Induced by High-Fat Diet in Rice Field Eel (*Monopterus Albus*)

**DOI:** 10.1155/2023/5533414

**Published:** 2023-03-16

**Authors:** Yajun Hu, Junzhi Zhang, Minglang Cai, Wuying Chu, Yi Hu

**Affiliations:** ^1^Hunan Engineering Technology Research Center of Featured Aquatic Resources Utilization, Hunan Agricultural University, Changsha Hunan 410128, China; ^2^College of Animal Science and Technology, Hunan Agricultural University, Changsha Hunan 410128, China; ^3^Department of Bioengineering and Environmental Science, Changsha University, Changsha Hunan 410000, China

## Abstract

An eight-week feeding trial explored the mechanism that supplemented methionine (0 g/kg, 4 g/kg, 8 g/kg, and 12 g/kg) in a high-fat diet (120 g/kg fat) on intestinal lipid transportation and gut microbiota of *M. Albus* (initial weight 25.03 ± 0.13 g) based on the diet (60 g/kg fat), named as Con, HFD+M0, HFD+M4, HFD+M8, and HFD+M12, respectively. Compared with Con, gastric amylase, lipase, trypsin (*P* < 0.05), and intestinal lipase, amylase, trypsin, Na^+/^K^+^ -Adenosinetriphosphatase, depth of gastric fovea, and the number of intestinal villus goblet cells of HFD+M0 were markedly declined (*P* < 0.05), while intestinal high-density lipoprotein-cholesterol, very low-density lipoprotein-cholesterol and microsomal triglyceride transfer protein of HFD+M0 were markedly enhanced (*P* < 0.05); compared with HFD+M0, gastric lipase, amylase, trypsin, and intestinal lipase, trypsin, Na^+^/K^+^ -Adenosinetriphosphatase, microsomal triglyceride transfer protein, very low-density lipoprotein-cholesterol, and apolipoprotein -A, the height of intestinal villus and the number of intestinal villus goblet cells of HFD+M8 were remarkably enhanced (*P* < 0.05). Compared with Con, intestinal *occ*, *cl12*, *cl15*, *zo-1*, *zo-2* of HFD + M0 were markedly down-regulated (*P* <0.05), while intestinal *vldlr*, *npc1l1*, *cd36*, *fatp1*, *fatp2*, *fatp6*, *fatp7*, *apo*, *apoa*, *apob*, *apof*, *apoo*, *mct1*, *mct2*, *mct4*, *mct7*, *mct12*, *lpl*, *mttp*, *moat2*, *dgat2* of HFD M0 were remarkably upregulated (*P* < 0.05); compared with HFD+M0, intestinal *gcn2* and *eif2α* of HFD+M8 were remarkably downregulated (*P* < 0.05), intestinal *occ*, *cl12*, *cl15*, *zo-1*, *zo-2*, *hdlbp*, *ldlrap*, *vldlr*, *cd36*, *fatp1*, *fatp2*, *fatp6*, *apo*, *apoa*, *apob*, *apof*, *apoo*, *mct1*, *mct2*, *mct8*, *mct12*, *lpl*, *mttp*, *moat2*, and *dgat2* were remarkably upregulated (*P* < 0.05). Compared with Con, the diversity of gut microbiota of HFD+M0 was significantly declined (*P* < 0.05), while the diversity of gut microbiota in HFD+M8 was significantly higher than that in HFD+M0 (*P* < 0.05). In conclusion, a high-fat methionine deficiency diet destroyed the intestinal barrier, reduced the capacity of intestinal digestion and absorption, and disrupted the balance of gut microbiota; supplemented methionine promoted the digestion and absorption of lipids, and also improved the balance of gut microbiota.

## 1. Introduction

Soybean protein is a high-quality plant protein and, with a short growth cycle, was widely used in aquatic feed to replace a fish meal [1]. However, methionine is the most lacking amino acid in soybean protein, it is also essential amino acid for fish [2]. There have been lots of studies reported that a higher proportion of soybean protein used in aquatic feed replacing fish meal inhibits growth performance and the body's metabolism of fish [3–5]. Additionally, methionine also regulates the body's metabolism with metabolites of methionine through transsulfuration [6], transamination [7], and transmethylation [8]. Besides, methionine can also be considered as a signal molecule induce various metabolism for aquatic animals [9–11]. Methionine restriction not only inhibits protein synthesis but also damages the healthy state of fish [12–14] and decreases intestinal digestive and antioxidant enzymes and immunomodulatory of rohu (*Labeo rohita*) [15].

The gastrointestinal tract is mainly involved in digestion and absorption. Gastrointestinal tract health and functional integrity have significant effects on an animal's overall healthy statement and utilization of nutrients, and gastrointestinal function includes digestion and absorption of nutrients by epithelial and goblet cells, secretion of immunoglobulins and mucins, also forming a barrier against harmful antigens and pathogens [16]. A study reported that branched-chain amino acid could promote intestinal morphology and cell breeding and enhance intestinal amino acid absorption by inducing intestinal amino acid transporters and promoting intestinal protein turnover [17]. Methionine also benefits intestinal morphology; besides, gut bacteria are related to extensive catabolism of dietary methionine in the intestine [18]. A previous study on nursery pigs indicated that dietary methionine improves small intestinal morphology by enhancing villous height and reducing bacteria fermentation via improving nutrient digestion and absorption [19]. What is more, supplement methionine could assist in producing glutathione and enhance the morphology of the duodenum of the nursery pig [20].

Gut microbiota could affect a host's lipid metabolism through multiple direct and indirect biological mechanisms [21], even considered as an endocrine organ [22]. Semova et al. [23] used fluorescent markers to image zebrafish (*Barchydanio rerio* var) and found that gut microbiota could stimulate the uptake of fatty acids and formation of lipid droplets by intestinal epithelium and liver, sclerenchyma enhances the absorption capacity of fatty acids by host's intestinal cells, thus promote to increase the amount of lipid droplets. Meanwhile, the size of lipid droplets was increased with the increasing abundance of bacteria, they demonstrated that the golden rod bacteria (*Chrysobacterium hispanicum*) and pseudomonas (*Pseudomonas adaceae*) are the main bacteria regulating the size of host lipid droplets. [24] reported that gut bacteria could ferment the indigestible carbohydrates, and digest the indigestible fiber of gut contents into short-chain fatty acids. Additionally, gut microbiota could promote the absorption of fatty acids by activating the absorption capacity of intestinal epithelial cells. Our previous studies showed that a high-fat (fish oil) diet disturbance the homeostasis of intestinal microbiota, which includes microbial population, an abundance of main microbiota, and an intestinal environment [25]. Meanwhile, dysbiosis of gut microbiota induced by dietary oxidized fish oil, while supplemented taurine could recover gut microbiota homeostasis and restore intestinal function [26]. In addition, the abundance of firmicutes of gut microbiota decreased with the supplementation of black soldier fly (*Hermetia illucens* L.) larvae meal [27].

Rice field eel (*Monopterus albus, M. albus*) is subtropical freshwater benthic fish [28], and prey on frog eggs, insects, earthworms, and water earthworms in nature [17]. *M. albus* has a gastrointestinal system, that needs better quality and higher levels of protein, and optimum protein/lipid ratio [29]. We found that when fish meal is replaced by soybean meal [30], soy protein concentrate [31] declines the growth performance of *M. albus*. Our earlier study showed that methionine deficiency inhibits the growth performance of *M. albus* [32, 33], also reduces bodies and hepatic lipid deposition, and mainly declined fatty acid synthesis [34]. Additionally, a deficiency of methionine damages gastric and intestinal structure reduces the function of the intestinal barrier and inhibits the ability of intestinal lipid and fatty acid transportation of *M. albus* (Hu et al., [32, 33]). What is more, a study reported that suitable methionine restriction improved gut function by regulating gut microbiota and its metabolite profiles in a high-fat diet on mice [35]. However, how gut microbiota adaptively change and regulate the host's metabolism under the condition of methionine restriction and methionine affect lipid transportation by gut microbiota remains unknown. Here, we made methionine deficiency experimental feed according to our previous study [32, 33]; additionally, made a high-fat diet based on our previous study that the high-fat model on *M. albus* [36]. We explored methionine and how to regulate intestinal lipid transportation and the gut microbiome of *M. albus*.

## 2. Materials and Methods

### 2.1. Experimental Feed

The basic feed (60 g/kg fat) was made according to our previous paper [32, 33], named Con; and the high-fat basic feed (120 g/kg fat) was based on our study on *M. Albus* [36], different levels of methionine (0, 4 g/kg, 8 g/kg, and 12 g/kg) were supplemented into high-fat diet obeyed the principle of equal nitrogen referenced our study [32, 33], named as, HFD+M0, HFD+M4, HFD+M8, and HFD+M12, respectively. Experimental diets and levels of nutrition is shown in Table 1.

The method of proximate analysis (moisture, crude protein, crude lipid, and ash) of experimental feed was referenced in our paper [37]. Amino acids were determined by an automatic amino acid analyzer (Agilent-1100, Agilent Technologies Co., Ltd., Santa Clara, CA, USA) referenced by the method reported by Wiriduge et al. [38], fatty acids were analyzed by GC-MS (Agilent 7890B-5977A, Agilent Technologies Co., Ltd., Santa Clara, CA, USA) referenced the method reported by Jin et al. [39], as shown in Tables 2 and 3.

### 2.2. Feeding Trial

The uniform size of *M. albus* (25.03 ± 0.13 g) was randomly raised in 15 cages (2.0 × 1.5 × 1.5 m), and every group contained triplicates with 60 fish, referenced to our study [32, 33]. Each cage was covered by 95% of the fresh alternanthera philoxeroides (Mart.) Griseb to simulate the natural environment for *M. albus*, water temperature 25 ± 6°C, dissolved O_2_ ≥ 6.0 mg/L, NH_4_^+^-N < 0.5 mg/L, respectively). The feeding rate was 3-4% of body weight and adjusted every two weeks. The feeding trial was lasting for 8 weeks.

### 2.3. Ethics Statement

Our study was approved by the Committee of Laboratory Animal Management and Animal Welfare of Hunan Agricultural University (Changsha, China) No. 094.

### 2.4. Sample Collection and Analyses

Stomach and intestine were obtained from six fish per cage, stored at -80°C until use. A gastric digestive enzyme (amylase, lipase, and trypsin) and intestinal biochemical indices (amylase, lipase, trypsin, Na^+^/K^+^ -Adenosinetriphosphatase, high-density lipoprotein cholesterol, and low-density lipoprotein cholesterol) were determined by a kit of NanJing JianCheng Bioengineering (Nanjing, China). Intestinal very-low-density lipoprotein cholesterol, microsomal triglyceride transfer protein, and Apolipoprotein-A were determined by a kit of Shanghai Enzyme-linked Biotechnology Co., Ltd (Shanghai, China).

The stomach and intestine of five fish in each cage were taken for observing organizational structure. Intestinal H&E-stained sections were referenced in our paper [40], and intestinal sections were made by cryostat microtome, stained with Oil Red O [32, 33], and observed by CaseViewer.

Intestinal total RNA was obtained from 6 fish per cage by Monzol reagent (Monad, Shanghai, China). RNA was reversed by MonScript (Monad, Shanghai, China) and obtained cDNA. Primers were synthesized by Biosune Biotechnology, Inc. (Shanghai, China), as shown in Table 4. Quantitative real-time PCR (qPCR) referenced our earlier paper [41]. The amplification efficiency was between 0.96 and 1.10, calculated by formula E = 10^∗^(−1/slope) − 1, and 5-fold serial dilutions of cDNA (triplicate) were used to generate the standard curve. Relative mRNA expression was calculated by 2^-△△Ct^ [42].

### 2.5. Gut Microbiota Analysis

Gut bacterial DNA was obtained from the gut content of three groups (Con, HFD+M0, and HFD+M8) by Power Fecal DNA Isolation Kit (MoBio Laboratories, Inc), and gut bacterial high-throughput sequencing by Illumina MiSeq platform, all sequences were classified into operational taxonomic units (OTUs) above the level of 97% similarity by quantitative insights into microbial ecology (QIIME) after removing low-quality scores (Q score, 20) with FASTX-Toolkit (Hannon Lab, USA).

### 2.6. Statistical Analysis

Data among groups (Con, HFD+M0, HFD+M4, HFD+M8, and HFD+M12) were analyzed by one-way analysis of variance (ANOVA), and remarkable differences among all groups were assessed by Turkey's multiple-range test. Values among groups (Con and HFD+M0; HFD+M0 and HFD+M8, respectively) were calculated by independent T-test by SPSS 22 software. Values were indicated as means ± SEM (standard error of the mean), and a significant difference was considered at *P* < 0.05.

## 3. Result

### 3.1. Gastric and Intestinal Biochemical Indices

Compared with Con, gastric amylase and trypsin were remarkably decreased in HFD+M0 (*P* < 0.05); intestinal lipase, amylase, trypsin, and Na^+/^K^+^ -Adenosinetriphosphatase were markedly decreased in HFD+M0 (*P* < 0.05), while intestinal high-density lipoprotein cholesterol, very-low-density lipoprotein cholesterol, and microsomal triglyceride transfer protein were significantly enhanced (*P* < 0.05). Compared with HFD+M0, dietary 8 g/kg methionine remarkably increased gastric lipase, amylase, and trypsin (*P* < 0.05), also significantly increased intestinal lipase, trypsin and Na^+^/K^+^ -Adenosinetriphosphatase, very-low-density lipoprotein cholesterol, microsomal triglyceride transfer protein, and Apolipoprotein-A (*P* < 0.05) (Table 5).

### 3.2. Gastric and Intestinal Sections

**Figure 1:**
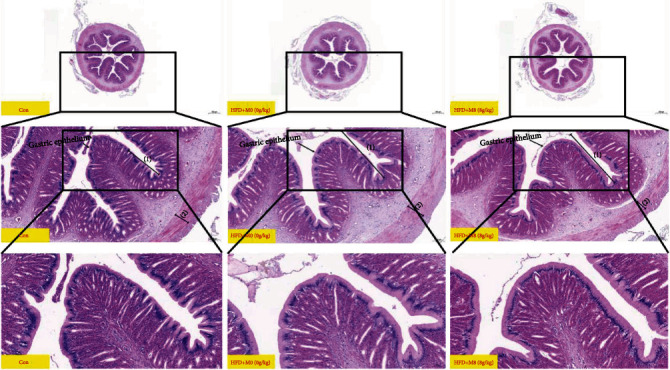
Effects of a high-fat diet supplemented with methionine on the gastric H&E-stained sections(1) and (2) were shown as gastric fovea and gastric muscular thickness, respectively.

**Figure 2:**
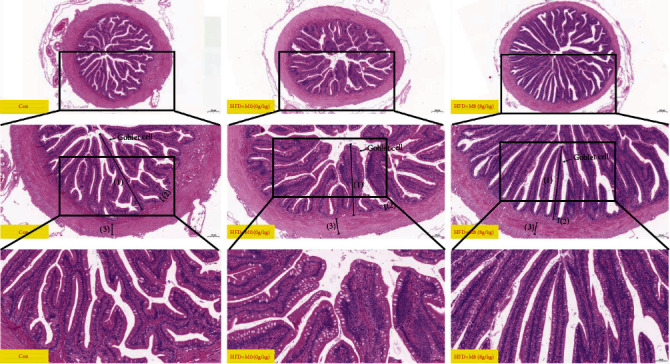
Effects of high-fat diet supplemented methionine on the intestinal H&E stained sections: (1), (2), (3), and (4) were shown intestinal villus height, crypt depth, intestinal muscular thickness, and intestinal goblet cell, respectively.

**Figure 3:**
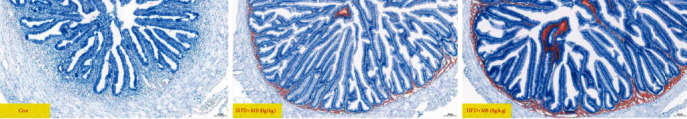
Effects of high-fat diet supplemented methionine on the intestinal oil red O stained sections (×100).

Compared with Con, gastric fovea remarkably decreased in HFD+M0 (*P* < 0.01), while dietary methionine increased gastric fovea (*P* > 0.05) (Figure 1, Table 6). Compared with Con, intestinal villus height and intestinal muscular thickness decreased in HFD+M0 (*P* > 0.05), and amounts of intestinal goblet cells per root in HFD+M0 were markedly decreased (*P* < 0.05), while dietary 8 g/kg methionine markedly improved intestinal villus height and goblet cells per root, remarkably decreased crypt depth (*P* < 0.05) (Figure 2, Table 7). Additionally, intestinal Oil Red O-stained sections showed that the content of lipid droplets of intestinal villi and the mucosal layer was as follows: Con < HFD+M0 < HFD+M8 (Figure 3).

### 3.3. Intestinal mRNA Expression

**Figure 4:**
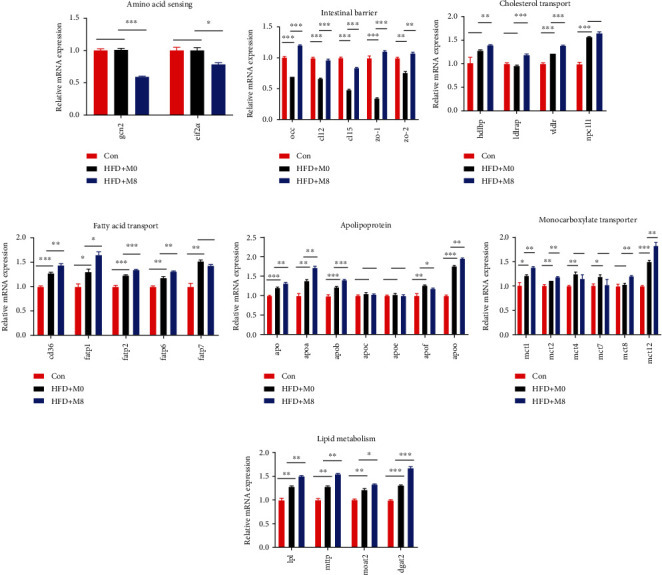
Effects of high-fat diet-supplemented methionine on intestinal mRNA expression (*n* = 3), ^∗^*P* < 0.05, ^∗∗^*P* < 0.01, and ^∗∗∗^*P* < 0.001.

Compared with Con, the intestinal *occ*, *cl12*, *cl15*, *zo-1*, and *zo-2* markedly downregulated in HFD+M0 (*P* < 0.001, *P* < 0.001, *P* < 0.001, *P* < 0.001, and *P* < 0.01, respectively), while intestinal *vldlr*, *npc1l1*, *cd36*, *fatp1*, *fatp2*, *fatp6*, *fatp7*, *apo*, *apoa*, *apob*, *apof*, *apoo*, *mct1*, *mct2*, *mct4*, *mct7*, *mct12*, *lpl*, *mttp*, *moat2*, and *dgat2* were markedly upregulated in HFD+M0 (*P* < 0.001, *P* < 0.001, *P* < 0.001, *P* < 0.05, *P* < 0.001, *P* < 0.01, *P* < 0.01, *P* < 0.001, *P* < 0.01, *P* < 0.001, *P* < 0.01, *P* < 0.001, *P* < 0.05, *P* < 0.01, *P* < 0.01, *P* < 0.05, *P* < 0.001, *P* < 0.01, *P* < 0.01, *P* < 0.01, and *P* < 0.001). Compared with HFD+M0, intestinal *gcn2* and *eif2α* downregulated in HFD+M8 (*P* < 0.001, *P* < 0.05), while intestinal *occ*, *cl12*, *cl15*, *zo-1*, *zo-2*, *hdlbp*, *ldlrap*, *vldlr*, *cd36*, *fatp1*, *fatp2*, *fatp6*, *apo*, *apoa*, *apob*, *apof*, *apoo*, *mct1*, *mct2*, *mct8*, *mct12*, *lpl*, *mttp*, *moat2*, and *dgat2* significantly upregulated (*P* < 0.001, *P* < 0.001, *P* < 0.001, *P* < 0.001, *P* < 0.01, *P* < 0.01, *P* < 0.001, *P* < 0.001, *P* < 0.01, *P* < 0.05, *P* < 0.001, *P* < 0.01, *P* < 0.01, *P* < 0.01, *P* < 0.001, *P* < 0.05, *P* < 0.01, *P* < 0.01, *P* < 0.01, *P* < 0.01, *P* < 0.01, *P* < 0.01, *P* < 0.01, *P* < 0.05, and *P* < 0.001) (Figure 4).

### 3.4. Gut Bacterial Diversity Indices

Compared with Con, the gut bacterial Chao1, OTUs, Shannon, Simpson, and Faith_pd in HFD+M0 were markedly declined (*P* < 0.001, *P* < 0.001, *P* < 0.001, *P* < 0.01, and *P* < 0.001). Compared with HFD+M0, gut bacterial Chao1, OTUs, Shannon, Simpson, and Faith_pd in HFD+M8 were significantly enhanced (*P* < 0.05, *P* < 0.05, *P* < 0.01, *P* < 0.05, and *P* < 0.001) (Table 8).

### 3.5. Gut Bacterial Levels of Phylum and Genus

**Figure 5:**
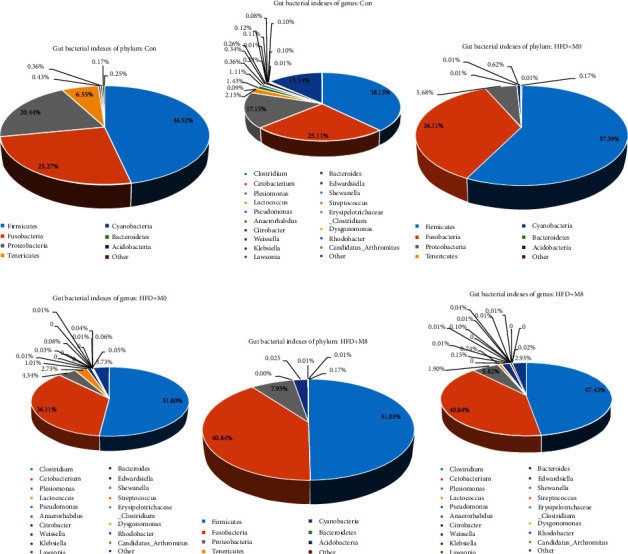
Effects of high-fat diet-supplemented methionine on gut bacterial indices of the phylum (a, c, e) and the genus (b, d, f) (*n* = 3).

Firmicutes, Fusobacteria, and Proteobacteria were the main populations at the phylum level, and Clostridium, Cetobacterium, and Plesiomonas were the main populations at the genus level. Compared with Con, the proportions of Firmicutes and Fusobacteria of the phylum and Clostridium and Cetobacterium of the genus were increased in HFD+M0, and the proportion of Proteobacteria of phylum and Plesiomonas of genus decreased. Compared with HFD+M0, Firmicutes of the phylum and Clostridium of the genus were decreased in HFD+M8, and the proportion of Fusobacteria and Proteobacteria of the phylum and Cetobacterium and Plesiomonas of the genus were increased in HFD+M8 (Figure 5).

### 3.6. Gut Bacterial Functional Classification of KEGG

**Figure 6:**
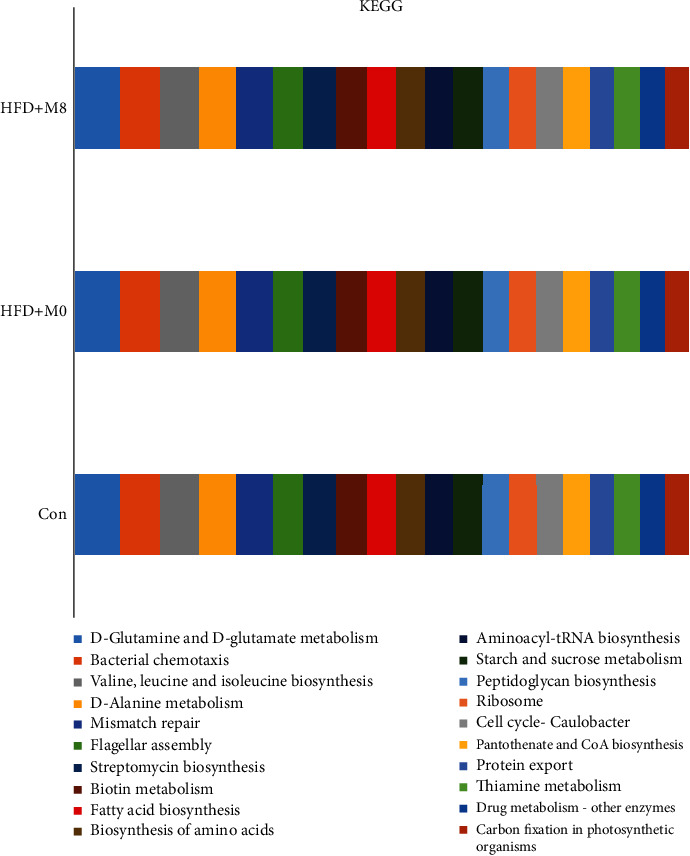
Effects of high-fat diet-supplemented methionine on gut bacterial COG function classification (*n* = 3).

The gut bacterial predictive main function was fatty acid biosynthesis, glutamine, glutamate, and alanine metabolism; valine, leucine, and isoleucine biosynthesis; and also including starch and sucrose metabolism (Figure 6).

## 4. Discussions

Recently, we found that dietary methionine restriction inhibits muscle fiber growth, development, and differentiation in *M. albus*, declines the growth performance in *M. albus* [32, 33], and induces hepatic lipid metabolism disorder and declined lipid content of *M. Albus* [34]. What is more, methionine deficiency also affected gastric and intestinal structure, damaged the intestinal barrier, and declined intestinal lipid and fatty acid transportation of *M. albus* [32, 33]. In this study, compared with the Con, gastric amylase and trypsin were remarkably decreased in the high-fat and methionine deficiency diet; also intestinal lipase, amylase, and trypsin were remarkably decreased in HFD+M0, while supplemented 8 g/kg methionine markedly increased gastric lipase, amylase, and trypsin, also markedly increased intestinal lipase and trypsin. From intestinal H&E-stained images, compared with Con, gastric fovea remarkably decreased in HFD+M0, while dietary methionine increased gastric fovea. Compared with Con, intestinal villus height and muscular thickness decreased in HFD+M0, and the number of intestinal goblet cells of root in HFD + M0 was markedly decreased, while dietary 8 g/kg methionine markedly improved intestinal villus height and goblet cells per root, remarkably decreased crypt depth. We inferred that a high-fat diet inhibited gastric-intestinal digestive capacity. Additionally, methionine sufficiency improved the gastric-intestinal main digestive enzymes (amylase, lipase, and trypsin) of *M. albus*, which is consistent with the study on grass carp (*Ctenopharyngodon idella*) [43].

MTTP promotes the transportation of fat by assisting triglyceride-rich apolipoprotein [44], such as ApoA1 [45]. HDL, LDL, and VLDL are major lipoproteins that carry cholesterol [46]. Na^+/^K^+^ -Adenosinetriphosphatase is the main enzyme involved in assisting the intestinal absorption and transportation of nutrients (such as amino acids, lipids, and glucose) [47]. Here, compared with Con, intestinal Na^+/^K^+^ -Adenosinetriphosphatase was remarkably decreased in HFD+M0, while intestinal HDL-C, VLDL-C, and MTTP were remarkably increased. Meanwhile, supplemented 8 g/kg methionine markedly increased intestinal Na^+^/K^+^ -Adenosinetriphosphatase, VLDL-C, MTTP, and ApoA. This indicated that an intestinal high concentration of fatty acids stimulated lipoprotein secretion [48], and dietary methionine could promote lipid transportation. This meant that the capacity of intestinal absorption declined, and the intestinal barrier was damaged [49].

Furtherly, we chose Con, HFD+M0, and HFD+M8 to explain the molecular mechanism of how methionine regulates gastrointestinal lipid digestion and absorption of *M. albus*. *gcn2* and *eif2a* can sense essential amino acid deprivation and regulates their downstream relative genes to adapt to nutrient deficiency [50]. In this study, compared with HFD+M0, intestinal *gcn2* and *eif2α* downregulated in HFD+M8, which meant that methionine deficiency could be sensed by *M. albus*. Occludens, claudin, and zonula are intestinal tight junction proteins, also important in protecting barrier integrity and preventing infiltration [51]. Here, compared with Con, the intestinal *occ*, *cl12*, *cl15*, and *zo-1*, *zo-2* remarkably downregulated in HFD+M0, dietary methionine markedly upregulated intestinal *occ*, *cl12*, *cl15*, *zo-1*, and *zo-2* expression. This explained that high-fat diet-induced free fatty acids damaged the intestine causes by which termed “intestinal lipotoxicity” [52], and methionine repaired the gastric and intestinal structure, also improved the intestinal barrier as we reported [32, 33]. MTTP promotes fatty transportation by assisting the secretion of lipoproteins [44]. *lpl* is involved in lipolysis [53], while *mogat2* and *dgat2* are the main enzymes involved in lipogenesis [54, 55]. In this study, compared with Con, the intestinal *lpl*, *mttp*, *moat2*, and *dgat2* were upregulated markedly in HFD+M0 upregulated, while dietary methionine significantly upregulated intestinal *lpl*, *mttp*, *moat2*, and *dgat2* expressions, which indirectly explained that why supplementing methionine enhanced gastric and intestinal digestive enzyme, also promoted the capacity of digestive. *dlbp* plays a pivotal role in the regulation of lipids and cholesterol [56], *ldlra* pathway is related to reducing circulating cholesterol [57]. *npc1l1* is mainly charged in cholesterol absorption [58]. *cd36* is related to fatty acid absorption and transportation [59, 60]. Apolipoprotein can bind and transport lipoproteins [61]. *mct* mainly transports short-chain monocarboxylates, including lactate, pyruvate, and ketone bodies [62]. Here, compared with Con, the intestinal *vldlr*, *npc1l1*, *cd36*, *fatp1*, *fatp2*, *fatp6*, *fatp7*, *apo*, *apoa*, *apob*, *apof*, *apoo*, *mct1*, *mct2*, *mct4*, *mct7*, *mct12*, *lpl*, *mttp*, *moat2*, and *dgat2* were significantly upregulated in HFD+M0. Compared with HFD+M0, intestinal *hdlbp*, *ldlrap*, *vldlr*, *cd36*, *fatp1*, *fatp2*, *fatp6*, *apo*, *apoa*, *apob*, *apof*, *apoo*, *mct1*, *mct2*, *mct8*, *mct12*, *lpl*, *mttp*, *moat2*, and *dgat2* remarkably upregulated. In addition, intestinal oil red O stained sections showed that the content of lipid droplets in intestinal villi, and the mucosal layer was as follows: Con < HFD+M0 < HFD+M8. We inferred that a high-fat diet damaged intestinal mucosal growth and reduces intestinal epithelial cells breeding, damaged the intestinal barrier [49, 63], then, increased intestinal permeability, intestinal lipid, and fatty acids transportation. While supplemented sufficiency methionine (8 g/kg) restores the intestinal barrier and improved intestinal function.

Compared with Con, the gut bacterial Chao1, OTUs, Shannon, Simpson, and Faith_pd in HFD+M0 were significantly decreased; compared with HFD + M0, the gut bacterial Chao1, OTUs, Shannon, Simpson, and Faith_pd in HFD+M8 were significantly increased. In addition, Firmicutes, Fusobacteria, and Proteobacteria were the main populations at the phylum level, and Clostridium, Cetobacterium, and Plesiomonas were the main populations at the genus level. What is more, compared with Con, the proportion of Firmicutes and Fusobacteria of the phylum and Clostridium and Cetobacterium of the genus were increased in HFD+M0, and the proportion of Proteobacteria of the phylum and Plesiomonas of the genus were decreased; compared with HFD+M0, Firmicutes of the phylum and Clostridium of the genus were decreased in HFD+M8, and the proportion of Fusobacteria and Proteobacteria of the phylum and Cetobacterium and Plesiomonas of the genus were increased in HFD+M8. We considered that a high-fat diet disturbed the balance of the bacterial community and induced microbial dysfunctions, the phenomenon was similar to our earlier study [25, 26], while supplemented methionine improved the gut microbiota homeostasis, and also promoted lipid metabolism. Meanwhile, the gut bacteria also had the predictive main function was fatty acid biosynthesis, glutamine, glutamate, and alanine metabolism; valine, leucine, and isoleucine biosynthesis; also including starch and sucrose metabolism.

In conclusion, the high-fat methionine deficiency diet affected the gastric and intestinal function of *M. albus*, damaged the intestinal barrier, reduced the capacity of intestinal digestion and absorption, and disrupted the balance of gut microbiota; supplemented methionine could improve the intestinal function, promoting the digestion and absorption of lipids, and also improving the gut microbiota balance.

## Figures and Tables

**Table 1:** Experimental diets and level of nutrition (%).

Ingredients	Con	HFD+M0	HFD+M4	HFD+M8	HFD+M12
Fish meal	11	11	11	11	11
Soy protein concentrate	40	40	40	40	40
Fish oil	4	10	10	10	10
^1^DL-methionine	0	0	0.4	0.8	1.2
Lysine	0.36	0.36	0.36	0.36	0.36
Glycine	1.6	1.6	1.2	0.8	0.4
Glutamate	0.4	0.4	0.4	0.4	0.4
^2^Attractant	0.1	0.1	0.1	0.1	0.1
Wheat meal	7.84	7.84	7.84	7.84	7.84
Cellulose	6	0	0	0	0
*α*-Starch	20	20	20	20	20
Brewer yeast	5	5	5	5	5
Choline chloride	0.5	0.5	0.5	0.5	0.5
Ca(H_2_PO_4_)_2_	2	2	2	2	2
^3^Vitamin and mineral premix	1.2	1.2	1.2	1.2	1.2
Total	100	100	100	100	100
Proximate analysis					
Dry matter (g/kg)	923.55	925.36	924.62	924.36	924.77
Crude protein (g/kg)	446.38	457.37	449.34	448.47	452.37
Crude lipid (g/kg)	67.95	126.26	127.37	127.72	126.13
Crude ash (g/kg)	101.16	100.36	102.32	101.25	100.47

^1^DL-Methionine (BR, 99%), ^2^attractant, and ^3^vitamin and mineral premix were referenced in our earlier study [32, 33].

**Table 2:** Contents of amino acids of experimental diets (g/kg).

Amino acids	Con	HFD+M0	HFD+M4	HFD+M8	HFD+M12
His^☆^	9.61	9.71	9.74	9.57	9.78
Ser	18.75	18.82	18.87	19.02	18.83
Arg^☆^	23.24	23.41	23.40	23.46	23.49
Gly	32.65	32.58	28.36	24.52	19.85
Asp	42.23	42.27	42.38	42.33	42.38
Glu	75.73	75.68	75.51	75.43	75.57
Thr^☆^	15.52	15.37	15.32	15.62	15.53
Ala	19.64	19.55	19.73	19.54	19.84
Pro	19.97	19.70	19.75	19.86	19.86
Cys	1.03	1.04	1.06	1.03	1.07
Lys^☆^	36.61	36.53	36.63	36.42	36.73
Tyr	9.88	9.70	9.78	9.79	9.84
Met^☆^	2.14	2.03	6.22	10.02	14.21
Val^☆^	18.53	18.41	18.37	18.74	18.53
Ile^☆^	17.23	17.43	17.52	17.32	17.10
Leu^☆^	29.64	29.73	29.71	29.86	29.52
Phe^☆^	18.43	18.78	18.53	18.44	18.41
Trp	/	/	/	/	/

^∗^Note: ☆for essential amino acids.

**Table 3:** Contents of fatty acids of experimental diets (mg/100 g).

Fatty acids	Con	HFD+M0	HFD+M4	HFD+M8	HFD+M12
C4:0	13.28	27.49	27.53	26.95	27.16
C8:0	5.17	10.21	10.05	10.04	10.2
C12:0	3.43	7.34	7.35	7.38	7.37
C13:0	11.14	22.17	21.32	22.46	22.74
C14:0	181.43	364.47	364.73	364.71	364.36
C14:1	2.43	5.12	5.08	5.2	5.13
C15:0	19.89	40.23	40.34	40.31	40.32
C16:0	603.12	1206.29	1209.21	1208.13	1206.77
C16:1	6.46	13.39	13.53	13.58	12.98
C17:0	13.4	26.63	26.75	26.34	26.24
C17:1	6.43	13.33	13.73	13.52	13.47
C18:0	120.35	241.39	241.87	241.88	241.73
18 : 1-T	16.23	32.84	32.53	32.46	32.71
C18:1 N-9C	411.25	818.96	819.39	818.85	819.58
18 : 2-T	2.74	5.75	5.73	5.64	5.96
C18:2 N-6C	16.35	33.71	33.34	32.85	33.12
C20:0	10.17	20.49	21.03	20.4	20.42
C20:1	26.84	53.47	53.49	53.86	54.95
C18:3 N-3	235.36	472.02	471.15	471.33	471.11
C20:2	10.72	20.91	20.89	20.93	21.04
C22:0	5.94	11.89	11.78	11.68	11.93
C22:1 N-9	198.23	394.76	395.38	395.69	394.88
C20:3 N-3	32.37	64.19	63.24	65.13	64.18
C20:4 N-6	25.25	50.49	50.54	50.36	50.51
C24:0	248.86	502.53	501.82	500.41	502.01
C20:5 N-3	101.37	201.56	201.71	201.88	201.83
C24:1	21.06	42.18	41.93	42.3	42.32
C22: 6 N-3	575.84	1143.39	1143.52	1145.11	1144.37

Note: C: Cis fatty acid; T: Transfatty Acids.

**Table 4:** Primer sequence for qPCR.

Gene	Forward (5′-3′)	Reverse (5′-3′)	^∗^Accession no.	Size (bp)
^1^ *gcn2*	GGAACTCGTCCTGAACTG	TGGTGAAGAACTTGCCTAT	XM_020586241.1	298
^2^ *eif2a*	CCCCTTCCTTTGTTCGTC	GCTGAGGCTTTCTTGTTCC	XM_020621840.1	121
^3^ *lpl*	CGTTGACATCGGAGACCTGA	CAAAGACCACCTTGGACTGAG	XM_020613041.1	146
^4^ *moat2*	TCTCCCTGCCTCTCTTTCA	TGTCCACTCCATAGTTGCCT	XM_020622089.1	213
^5^ *dgat2*	ACTTCCGCTTTCCCTTG	ATTCCCTGTCTCGTTATGTG	XM_020622054.1	104
^6^ *mttp*	AAGATGCTCCAGGCTTTGTT	TGTCAGGACCCTCTAAAATCAG	XM_020602163.1	172
^7^ *hdlbp*	CCACCCCAGACGACAAAGAC	GGCGAGCAACAAAATAACGA	XM_020609988.1	165
^8^ *ldlrap*	CAGGAAGACAAAAGCAAGAAGG	CGAGTGGGGTTACTATGAGGC	XM_020617284.1	194
^9^ *vldlr*	ACATCCGTCGTTTGGGTCTA	GTGGTAGTGTCCCCTCGTTT	XM_020601062.1	169
^10^ *npc1l1*	TTGGAGTCCCAGTTTATTT	TACACTTGCGTCCACATT	XM_020590431.1	297
^11^ *mct1*	TCCTATGCCTTCCCTAAAT	AAGTTGAATGCCAGTCCC	XM_020586598.1	287
^12^ *mct2*	TGGGCTTGTCACCATTAT	CTCCTCGTCCAGTTTCTT	XM_020589687.1	181
^13^ *mct4*	GAGGAGCAGTGGTGGATG	GGGAAGGCGTAGGAGAAA	XM_020608921.1	112
^14^ *mct7*	GTTGTCATTGGCACCCTT	ACCTGAGTCCTCCGAACC	XM_020608232.1	210
^15^ *mct8*	CAGCAGGACCTTCCAAAT	AAAGTAGCCCAGGACAGC	XM_020592011.1	271
^16^ *mct12*	GTTGGCGTATGGGATTGC	TTTGGCGAGATTTGGATGT	XM_020616669.1	223
^17^ *cd36*	TTGAAAGGGATTGAGGTG	TCTCGCAAGGATGGACTA	XM_020616796.1	212
^18^ *fatp1*	GCGAGCCAGGTATGTTAG	CAGCAAGGCACTGAGGAC	XM_020587461.1	263
^19^ *fatp2*	CTTTGATTACAGCCTTGC	CTTTCCGTTGTCCTTTCT	XM_020602138.1	100
^20^ *fatp6*	CAGTAGGACTTTGGGCATTT	GTCGCACTTTGTGAACTTTATC	XM_020618747.1	267
^21^ *fatp7*	ACTGTAATCATCAGCCAAGA	GGTTTCGTCAAACTCCTC	XM_020588511.1	105
^22^ *apo*	GGGCTGCTCTGGATGTCT	CCCGCAAAGCACTAATCT	HQ603782.1	147
^23^ *apoa*	CAAGAAGGTCCAGGTTGA	TTAGTAAGGGATTGGTAGAGG	XM_020590134.1	147
^24^ *apob*	TGCCAATAACTATCCGCTAC	TCTTCCTGACATCATCCC	XM_020615697.1	247
^25^ *apoc*	GCTGCTGGTCGTTACTGT	AGTCCCTAATGGTTTCTATG	XM_020590135.1	176
^26^ *apoe*	CGCTGCGTGGAAGGAAAC	CTGCCAGAGCAAGGATGAGA	XM_020590131.1	220
^27^ *apof*	AGGTGGTAAGCCTGATAGA	CCAACCCTCATAGTGTCC	XM_020592847.1	185
^28^ *apoo*	GCTCAGGTTCGGTTTGTT	GGTGGCAACTCTGGGTAT	XM_020595548.1	207
^29^ *occ*	TGTCGGGGAGTGGGTAAA	TCCAGGCAAATAAAGAGGCT	XM_020616177.1	130
^30^ *zo-1*	GGCATCATCCCCAACAAA	GCGAAGACCACGGAACCT	XM_020621576.1	111
^31^ *zo-2*	AGCCGAGGTCGCACTTTA	GCTTTGCTTCTGTGGTTGAT	XM_020615114.1	246
^32^ *cl-12*	TCACCTTCAATCGCAACG	ATGTCTGGCTCAGGCTTATCT	XM_020607277.1	250
^33^ *cl-15*	CTCGCTGCTTGCTTTGACT	TTGAAGGCGTACCAGGACA	XM_020611334.1	225
^34^ *rpL17*	CGAGAACCCGACTAAATCA	GTTGTAGCGACGGAAAGG	XM_020587712.1	169

^1^ *gcn2*: general control nonderepressible; ^2^*eif2a*: eukaryotic translation initiation factor 2; ^3^*lpl*: lipoprotein lipase; ^4^*moat2*: monoacylglycerol O-acyltransferase 2; ^5^*dgat2*: diacylglycerol acyltransferase 2; ^6^*mttp*: microsomal triglyceride transfer protein; ^7^*hdlbp*: high-density lipoprotein binding protein; ^8^*ldlrap*: low-density lipoprotein receptor adapter protein; ^9^*vldlr*: very low-density lipoprotein receptor; ^10^*npc1l1*: NPC1 like intracellular cholesterol transporter 1; ^11^*mct1*: monocarboxylate transporter 1-like; ^12^*mct2*: monocarboxylate transporter 2-like; ^13^*mct4*: monocarboxylate transporter 4-like; ^14^*mct7*: monocarboxylate transporter 7-like; ^15^*mct8*: monocarboxylate transporter 8-like; ^16^*mct12*: monocarboxylate transporter 12-B-like; ^17^*cd36*: CD36 molecule; ^18^*fatp1*: fatty acid transport protein 1; ^19^*fatp2*: fatty acid binding protein 2; ^20^*fatp6*: fatty acid transport protein 6; ^21^*fatp7*: fatty acid binding protein 7; ^22^*apo*: apolipoprotein; ^23^*apoa*: apolipoprotein A; ^24^*apob*: apolipoprotein B; ^25^*apoc*: apolipoprotein C; ^26^*apoe*: apolipoprotein E. ^27^*apof*: apolipoprotein F; ^28^*apoo*: apolipoprotein O; ^29^*occ*: Occludin-like; ^30^*zo-1*: tight junction protein ZO-1-like; ^31^*zo-2*: tight junction protein ZO-2-like; ^32^*cl-12*: Claudin 12; ^33^*cl-15*: Claudin 15; ^34^*rpL17*: ribosomal protein L17, it is reference gene. ^∗^NCBI reference sequence.

**Table 5:** Effects of high-fat diet supplemented different levels of methionine on gastric and intestinal biochemical indices.

Index	Con	HFD+M0	HFD+M4	HFD+M8	HFD+M12	*P* value
Stomach						
^1^Lip	422.47 ± 16.44^ab^	348.79 ± 22.08^a^	466.69 ± 20.73^bc^	550.2 ± 33.75^c^	535.46 ± 25.71^c^	<0.001
^2^Amy	215.62 ± 4.03^c^	164.06 ± 3.12^a^	167.53 ± 1.69^a^	195.58 ± 6.73^b^	195.05 ± 3.67^b^	<0.001
^3^Try	2110.3 ± 90.1^b^	1666.38 ± 100.43^a^	1777.93 ± 87.84^ab^	2078.42 ± 64.78^b^	2007.85 ± 53.95^b^	0.002
Intestine						
^1^Lip	324.5 ± 6.31^b^	272.77 ± 5.95^a^	366.83 ± 12.62^b^	423.26 ± 16.29^c^	427.97 ± 13.47^c^	<0.001
^2^Amy	251.62 ± 9.04^c^	165.11 ± 5.1^a^	175.83 ± 4.21^ab^	181.19 ± 9.17^ab^	195.48 ± 4.54^b^	<0.001
^3^Try	2175 ± 30.88^b^	1634.52 ± 55.78^a^	1952.71 ± 70.79^b^	1965.78 ± 60.36^b^	1941.81 ± 72.86^b^	<0.001
^4^Na^+^/K^+^-ATP	342.25 ± 7.67^bc^	204.17 ± 7.81^a^	309.2 ± 30.77^b^	412.47 ± 10.82^c^	410.7 ± 6.38^c^	<0.001
^5^HDL-C	9.64 ± 0.31^a^	12.13 ± 0.64^bc^	10.57 ± 0.39^ab^	13.99 ± 0.42^c^	11.5 ± 0.89^ab^	<0.001
^6^LDL-C	48.02 ± 2.43	54.92 ± 2.91	57.1 ± 4.16	53.83 ± 3.73	58.92 ± 3.27	0.224
^7^VLDL-C	120.4 ± 1.11^a^	134.28 ± 0.79^b^	129.99 ± 2.74^b^	141.86 ± 1.02^c^	159.5 ± 1.82^d^	<0.001
^8^MTTP	43.87 ± 0.61^a^	52.96 ± 0.45^b^	64.37 ± 0.85^d^	65.32 ± 1.31^d^	60.18 ± 0.67^c^	<0.001
^9^ApoA	65.27 ± 3.87^a^	64.1 ± 5.54^a^	71.92 ± 3.6^ab^	88.91 ± 6.39^b^	83.33 ± 5.11^ab^	0.005

Note: values are presented as means ± SEM (*n* = 3). Letters in the same row with the same superscript or absence of superscripts are not a significant difference (*P* > 0.05). ^1^Lip: Lipase (U/g protein); ^2^Amy: Amylase (U/g protein); ^3^Try: Trypsin (U/g protein); ^4^Na^+^/K^+^-ATP: Na^+^/K^+^ -Adenosinetriphosphatase (mmolPi/mg prot/hour); ^5^HDL-C: High-density lipoprotein cholesterol (mmol/g prot); ^6^LDL-C: low-density lipoprotein cholesterol (mmol/g prot); ^7^VLDL-C: very low-density lipoprotein cholesterol (mmol/g prot); ^8^MTTP: Mirosomal triglyceride transfer protein (pg/mg prot); ^9^Apo-A: Apolipoprotein -A (ug/g prot).

**Table 6:** Effects of a high-fat diet supplemented with methionine on the gastric H&E-stained sections (100 times and 400 times).

Index	Con	HFD + M0	HFD + M8	P value	
				^∗^	#
^1^GF	673.41 ± 29	541.53 ± 21.1^∗∗^	602.85 ± 29.31	0.004	0.12
^2^GMT	122.1 ± 3.39	114.96 ± 3.47	121.07 ± 4.32	0.172	0.296

The values are displayed as means ± SEM (*n* = 3). The ^∗^ expressed that there is a significant difference between the Con and HFD+M0 groups, and the ^#^expressed that there is a significant difference between the HFD+M0 and HFD+M8 groups. The same below, ^1^GF: gastric fovea (*μ*m); ^2^GMT: gastric muscular thickness (*μ*m).

**Table 7:** Effects of high-fat diet supplemented methionine on the intestinal H&E-stained sections (100 times and 400 times).

Index	Con	HFD+M0	HFD+M8	*P* value	
				^∗^	#
^1^IVH	594.57 ± 39.59	508.66 ± 20.05	638.69 ± 31.46^##^	0.082	0.006
^2^CD	24.72 ± 1.58	25.96 ± 1.49	20.25 ± 1.45^#^	0.580	0.021
^3^IMT	105.1 ± 3.2	104.57 ± 3.33	95.62 ± 3.51	0.911	0.094
^4^AIGC	36 ± 1	31 ± 1^∗^	38 ± 3^#^	0.020	0.046

^1^IVH: intestinal villus height (*μ*m); ^2^CD: crypt depth (*μ*m); ^3^IMT: intestinal muscular thickness (*μ*m); ^4^AIGC: amounts of intestinal goblet cells per root.

**Table 8:** Effects of high-fat diet supplemented methionine on gut bacterial diversity indices (*n* = 3).

Index	Con	HFD + M0	HFD + M8	P value	
				^∗^	#
Chao1	213.47 ± 19.72	55.25 ± 4.42^∗∗∗^	80.04 ± 6.96^#^	<0.001	0.013
OTUs	212 ± 19	55 ± 4^∗∗∗^	80 ± 6^#^	<0.001	0.013
Shannon	3.6 ± 0.23	1.85 ± 0.08^∗∗∗^	2.2 ± 0.06^##^	<0.001	0.008
Simpson	0.77 ± 0.05	0.52 ± 0.03^∗∗^	0.62 ± 0.02^#^	0.001	0.015
Faith_pd	19.74 ± 2.35	7.49 ± 0.47^∗∗∗^	16.1 ± 0.91^###^	<0.001	<0.001

## Data Availability

The datasets used and/or analyzed during the current study are available from the corresponding author upon reasonable request.
